# A Systematic Review of Peripheral and Central Nervous System Involvement of Rheumatoid Arthritis, Systemic Lupus Erythematosus, Primary Sjögren's Syndrome, and Associated Immunological Profiles

**DOI:** 10.1155/2015/910352

**Published:** 2015-11-25

**Authors:** Anastasia Bougea, Evangelos Anagnostou, Giatas Konstantinos, Paraskevas George, Nikolaos Triantafyllou, Evangelia Kararizou

**Affiliations:** 1st Department of Neurology, University of Athens Medical School, Eginition Hospital, Athens, Greece

## Abstract

Both central (CNS) and peripheral nervous system (PNS) complications are frequent and varied in connective tissue diseases. A systematic review was conducted between 1989 and 2014 in the databases Medline, Scopus, and Cochrane Library using the search terms, peripheral and central nervous complications and immunological profiles, to identify studies in specific connective tissue disorders such as rheumatoid arthritis, systemic lupus erythematosus, and primary Sjögren's syndrome. A total of 675 references were identified, of which 118 were selected for detailed analysis and 22 were included in the final review with a total of 2338 participants. Our search focused only on studies upon connective tissue disorders such as rheumatoid arthritis, systemic lupus erythematosus, and primary Sjögren's syndrome associated with seroimmunological data. The reported prevalence of CNS involvement ranges from 9 to 92% across the reported studies. However, the association between CNS and PNS manifestations and seroimmunological profiles remains controversial. Τo date, no laboratory test has been shown as pathognomonic neither for CNS nor for PNS involvement.

## 1. Introduction

Connective tissue disorders are chronic inflammatory autoimmune diseases driven by an antibody or T-cell response directed against a self-antigen antibody affecting muscle, joints, and skin such as rheumatoid arthritis (RΑ), systemic lupus erythematosus (SLE), primary Sjögren's syndrome (pSS), and systemic sclerosis [1–3]. Both immune mediated changes in the vasculature of the vessels walls, as a hallmark of vasculitis, may be associated with central nervous system (CNS) and peripheral nervous system (PNS) symptoms. The vascular injury may be related to the presence of antibodies most commonly, but not limited to, SS or a related profile of autoantibodies including antinuclear antibody (ANA), extractable nuclear antigen antibodies (SSA/SSB), rheumatoid factor (RF), anticardiolipin antibodies (ACA), cryoglobulins, and anti-double-stranded DNA antibody (A-ds DNA) [4] (Table 1). Anti-ribosomal P antibodies have been associated with CNS SLE disease [5].

To our knowledge this is the first systematic review focused in both most common CNS and PNS complications of RA, SLE, and pSS emphasizing the associated immunological features of these specific connective tissue.

## 2. Methods

### 2.1. Eligibility Criteria and Source Selection

This is a systematic review of literature, based on the PRISMA guidelines. We search for relevant studies in English in the following databases, from the emergence of the condition to October 2014: Medline (from 1989 to 2014), Scopus (from 1983 to 2012), and Cochrane Library (from 1993 to 2014). The keywords used for the study were the following: “peripheral nervous and central nervous manifestations,” “myositis,” “cranial neuropathy,” “mononeuropathy,” “polyneuropathy,” “myelopathy,” “myelitis,” “multiple sclerosis,” “Neuromyelitis optica spectrum (NMO),” “headaches,” “seizures,” “psychosis,” “depression,” “connective tissue disorders,” “rheumatoid arthritis, systemic lupus erythematosus, primary Sjögren's syndrome,” “Immunological profiles,” “ANA,” “anti-ribosomal P antibodies,” “Antiphospholipid antibodies,” “SSA/SSB,” “RF,” “ACA,” “cryoglobulins,” “A-ds DNA,” and “AQ4 antibody”.

### 2.2. Inclusion and Exclusion Criteria

Articles not published in English, letters, summaries, dissertations, theses, and case reports were excluded, as well as studies that used children or animal models and studies that were not related to central and peripheral nervous complications of RA, SLE, and pSS. Studies were included only if diagnosis of neurological complications was made by expert neurologists. Only studies with neurological complications primary or secondary to RA, SLE, and PSS with associated immunological features were included. Immunological tests (i.e., gamma globulins, ANA, anti-ribosomal P antibodies, anti-Ro/SSA and anti-La/SSB antibodies, RF, AQ4 antibody, and cryoglobulins) were included if they were performed at the time of diagnosis and at least yearly during follow-up.

## 3. Results

**Figure 1:**
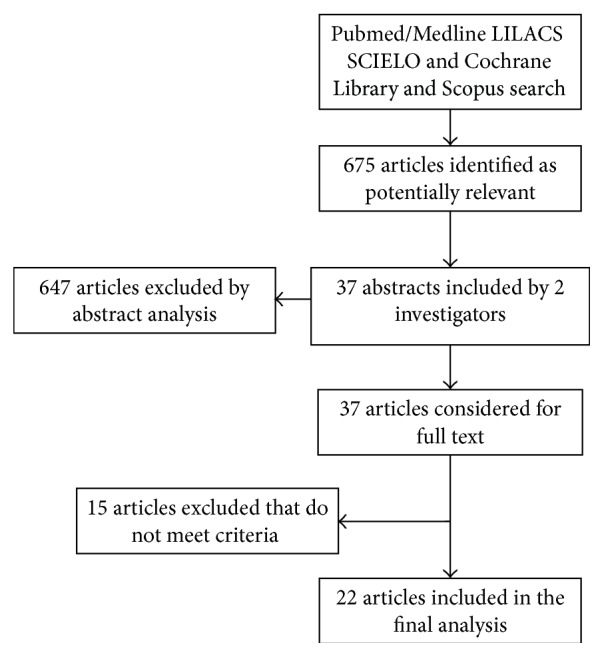
Research process flowchart.

The described search identified six hundred seventy-five relevant studies (Figure 1), 107 of which were excluded based on abstract analysis. The remaining 37 studies were reviewed on its full text and 22 were included in this systematic review that addressed the inclusion criteria (a total of 2338 patients). The majority of them were cohort studies (18); 1 was case-control and 1 was randomized controlled trial. Of these, 12 studies focused on SLE and 9 on SS. Only one study was found regarding patients with RA. Classification of each connective tissue disorders was assessed according to established criteria for SLE [6], SS [7], and RA [8].

We performed qualitative data synthesis, organizing the results by connective tissue type. We did not attempt to perform meta-analysis because of the heterogeneity of the study designs, populations, and results. Using our critical appraisal of individual studies and the body of evidence for each study design, we identified strengths and weaknesses of each study in the discussion. We did not assess publication bias.  Table 2 summarizes the main methodologic characteristics and results of the included studies.

In 1989, Mellgren et al. [29] reviewed 33 cases of primary SS and peripheral neuropathy evaluated at the Mayo Clinic from 1976 to 1988 and studied sural nerve biopsy specimens in 11 of them. Based on analytical clinical and electrophysiological (electromyography and sensory and motor conduction study) nerve features, 23 patients had PNS features such as distal sensorimotor neuropathy, 10 had a sensory neuropathy, five had trigeminal neuropathy, and two had carpal tunnel syndrome. The authors did not report any correlation between neuropathy and laboratory findings such as anti-SS-A/Ro antibodies, cryoglobulins, and RF in patients with SS.

In Andonopoulos et al.'s study [30], 10 of the 14 patients with evidence of PNS disease had a mild peripheral neuropathy of the glove and stocking distribution; six of them were mixed and four were sensory only. Two of those, plus one more, had trigeminal neuropathy. Two patients had abnormal terminal latencies of the peroneal nerves, indicative of motor neuropathy. Six of our primary SS subjects had skin vasculitis, all anti-SS-A/Ro positive. Two of those, with cryoglobulinemia and low C, had PNS disease (one with mononeuritis multiplex). Only one patient with severe primary SS, manifested by cryoglobulinemia, vasculitis, and glomerulonephritis, presented with mononeuritis multiplex that partially responded to intravenous cyclophosphamide and high-dose steroids. Of interest is the absence of any statistically significant correlation between the observed PNS pathology and any other clinical or immunologic feature.

Among 33 RA patients, Sivri and Güler-Uysal [28] found 2 (6%) patients to have carpal tunnel syndrome, while 6 (18%) patients had mononeuritis multiplex. Impaired nerve conduction velocities were detected in 6 (18%) of 33 RA patients suggesting CNS involvement with intact PNS (however, there is no information about CNS involvement). Of note, there was not any clear correlation between neuropathy and clinical or laboratory data (RF).

Kasitanon et al. [12] studied 91 patients (90 females, 1 male) fulfilling standardized criteria of ACR nomenclature for neuropsychiatric SLE (NPSLE) [31]. CNS manifestations included seizures in 53 patients (54.1%), psychosis in 13 (13.3%), acute confusion state in 11 (11.2%), abnormal consciousness in 6 (6.1%), transverse myelitis in 6 (6.1%), cerebral infarction in 2 (2.0%), and aseptic meningitis in 2 (2.0%). PNS manifestations such as peripheral neuropathy were reported in 5 (5.1%) patients. However, patients with NPSLE had significantly more cutaneous vasculitis than those without associations between NPSLE and immunoserological data.

In Schneebaum et al. study [9], 19% of 269 patients with SLE demonstrated elevated levels of IgG or IgM anti-P antibodies, including 14% of 187 patients without and 29% of 82 patients with neuropsychiatric manifestations. The frequency in patients with severe depression (*n* = 8) and psychosis (*n* = 29) was 88% and 45%, respectively, compared with only 9% in patients with nonpsychiatric neurologic disease (*n* = 45). For the entire SLE group, the odds ratio for the association of anti-P antibodies and severe psychiatric manifestations was 7.63 with a 95% confidence interval of 3.61 to 16.14.

Isshi and Hirohata [10] also analyzed sera from 87 SLE patients (27 with non-CNS SLE, 34 with lupus psychosis, and 26 with nonpsychotic CNS lupus) and from 20 control patients with neurologic manifestations without SLE and cerebrospinal fluid (CSF) from 41 patients with CNS lupus and from the 20 control patients for IgG anti-P. Serum anti-P levels were significantly elevated in patients with lupus psychosis compared with those with non-CNS SLE or those with nonpsychotic CNS lupus, indicating that anti-P in the systemic circulation are involved in the development of lupus psychosis.

In Tzioufas et al. study [11], serum was obtained from 28 SLE patients during active CNS involvement. Eleven patients had diffuse CNS involvement (seven had psychiatric disorders and four had grand mal seizures), 13 patients had focal CNS involvement, seven of whom were associated with antibodies to cardiolipin, and four patients had a mixed form of CNS disease. The overall prevalence of anti-P antibodies in active CNS disease patients was statistically and significantly higher, as compared to unselected SLE patients (*χ* ^2^ = 6.04, *P* < 0.05). These antibodies are associated with active SLE and CNS involvement particularly in patients without anticardiolipin antibodies.

In Sanna et al. study [13], 185 SLE patients (57.3%) had NP manifestations at any time during follow-up. Headache was the most frequent manifestation, present in 78 patients (24%). Cerebrovascular disease (CD) was diagnosed in 47/323 patients (14.5%), with a total of 57 events. Mood disorders were found in 54 (16.7%), cognitive disorders in 35 (10.8%), and seizures in 27 patients (8.3%). Psychosis was diagnosed in 25 (7.7%), anxiety disorder in 24 (3.7%), and acute confusional state in 12 patients (3.7%). NP manifestations are significantly associated with aPL. CD, headache, and seizures were independently associated with these antibodies.

In Hirohata et al. study [20], 23 patients showed that CNS manifestations other than diffuse NPSLE, including neurologic syndromes and PNS involvements (focal NPSLE), according to the ACR nomenclature for NPSLE anti-Sm levels in CSF and sera were measured using enzyme-linked immunosorbent assay (ELISA) kits. Anti-Sm in CSF were significantly elevated in NPSLE compared with non-SLE control. Among subsets of NPSLE, CSF anti-Sm was significantly elevated in acute confusional state compared with nonacute confusional state diffuse NPSLE (*P* = 0.0028) or with focal NPSLE (*P* = 0.0008).

In Závada et al. study [15], sera of 76 patients with SLE and neurological symptoms, 50 of whom met the ACR nomenclature for NPSLE, were tested for AQP4-Ab in an indirect immunofluorescence assay employing HEK293 cells transfected with recombinant human AQP4. Only one of the examined sera was positive for NMO-IgG/AQP4-Ab. None of the 75 NPSLE without was found to be seropositive for NMO-IgG/AQP4-Ab. NMO-IgG/AQP4-Ab in NPSLE were present only in a patient with transverse myelitis and were not detectable in NPSLE patients with other neurological manifestations.

Hanly et al. [16] identified 41 NPSLE patients. CNS manifestations accounted for 92% of the events compared to involvement of the PNS in 8%. In patients with NP, the occurrence of renal disease was significantly associated with use of prednisone and immunosuppressive drugs. However, associations between positive antinuclear antibodies (97%) and elevated anti-dsDNA antibodies were not examined.

According to 1997 American College of Rheumatology diagnostic criteria [6], 15 women were enrolled in the study of Kluz et al. [17] with severe disease activity (a modified SLE Disease Activity Index (SLEDAI) of >12 points) and inflammatory microangiopathy-related complications such as systemic CNS affection and/or vasculitis and/or nephritis. In 6 of them, CNS complications were seizure (*n* = 1), organic brain syndrome (*n* = 2), cranial nerve disorder (*n* = 1), and headache (*n* = 2). Significantly higher levels of both fractions of ACA and IgM ACA and of both fractions of ACA and IgM ACA and antiphospholipid antibodies (repeatedly positive test for serum ACA and/or lupus anticoagulant) were determined in patients with severe disease activity and microangiopathic complications compared with those with less active disease.

Briani et al. [18] presented 85 SLE patients (39%) with neurological manifestations. Both central (53 patients) and peripheral (23 patients) nervous systems were involved (Table 2). In 9 patients CNS and PNS manifestations concurred. The most common CNS manifestation was headache (35 cases, 58%) followed by cerebrovascular diseases (11 cases, 17%), epilepsy (10 cases, 16%), psychiatric disorders (6 cases, 10%), and myelopathy (2 cases, 3%). PNS manifestations included symmetric polyneuropathy (17 cases, 20%), mononeuropathy (13 cases, 10 with median nerve and 3 with sciatic nerve involvement, 35%), and mononeuropathy multiplex (2 cases, 5%). One patient with mononeuropathy multiplex underwent sural nerve biopsy, which showed vasculitic axonal injury. Of the 7 sera and CSF samples with Abs to ribosomal P proteins, these autoantibodies are associated with CNS manifestations such as psychosis (*P* = 0.017) and PNS mononeuropathy multiplex (*P* = 0.040).

Gono et al. [32] enrolled 20 patients with pSS. PNS involvement regarded seven patients with cranial neuropathy, nine patients with polyneuropathy, and three patients with mononeuritis multiplex (Table 2). Seven patients with cranial neuropathy included three with optic neuritis, two with trigeminal neuralgia, one with facial nerve palsy, and one with glossopharyngeal and vagus nerve palsy. Eight of the nine patients with polyneuropathy revealed pure sensory neuropathy. Two of the three patients with mono neuritis multiplex revealed motor sensory neuropathy. CNS involvement included three patients with encephalopathy and two patients with aseptic meningitis. One patient with optic neuritis had spinal cord lesions and cerebral focal symptoms, such as right hemiparesis, which are similar findings in multiple sclerosis. Moreover, AQ4 antibody was detected in the other one of the three patients with optic neuritis. This patient was diagnosed with Neuromyelitis optica (NMO) associated with pSS.

Among 207 SLE patients Florica et al. [19] showed that 125 had SLE-related PN involvement and 82 had non-SLE-related PN. The most frequent etiologies of non-SLE-related PN were compressive neuropathy (nerve or root compression) in 35 (42.6%), followed by medication toxicity in 23 (28%), hypothyroidism in 19 (23.1%), and diabetes mellitus in 14 (17%). Other causes of non-SLE-related PN included ethanol abuse, paraproteinemia, uremia, and viral hepatitis in a limited number of patients.

Patients with PNS were more likely to have also CNS involvement (14.2%) compared with patients without PN (CNS involvement: 6.6%, *P* = 0.02). The most common PN observed was peripheral polyneuropathy, with a predominance of the sensory form, present in 76 (36.7%) patients and the sensory-motor variant in 39 (18.8%) patients. Twenty-three (11.1%) patients had a peripheral mononeuropathy and 26 (12.5%) had a cranial neuropathy. Nineteen (9.2%) patients suffered from mononeuritis multiplex and only 11 (5.3%) were diagnosed with chronic inflammatory demyelinating polyradiculoneuropathy (CIDP). There was no difference between PNS features and SLE immune markers (presence of antiphospholipid antibodies).

In study of Chiewthanakul et al. [14], CNS manifestations accounted for 87% (84/97 patients), while involvement of the PNS was 13% (13/97 patients). The three most frequent CNS manifestations included seizures (33%), psychoses (22.7%), and cerebrovascular disease (22.3%), composed of 82.6% cerebral infarction, 8.7% transient ischemic attack, and 8.7% venous sinus thrombosis. ANA and antibodies to dsDNA did not correlate with NP manifestations.

In 2014 study by Jamilloux et al. [26], 420 patients fulfilled the 2002 American-European pSS criteria [7]. Within 93 (22%) patients with neurological manifestations, PN and CNS were involved in 66% and 44%, respectively. The number of extraglandular manifestations was increased in patients with sensorimotor neuropathies (3.3 ± 1.9 versus 2 ± 1.5, *P* < 0.05). This subgroup had more frequent cryoglobulinemia and lymphopenia (*P* < 0.05) but lower prevalence of anti-Ro/SSA antibodies and hypergammaglobulinemia (*P* < 0.05). Authors also confirmed cryoglobulinemia as unique predictive factor of PNS disease.

In Spezialetti et al. study [21], from 77 patients with pSS and CNS involvement, psychiatric or cognitive impairment, usually mild or moderate, occurred in over 80% (63 of 77) of this highly selected population of SS patients, and more than 60% of patients (48 of 77) had both. Anti-ribosomal P antibodies occurred in six (4.6%) patients with SS and related disorders. None of the patients with primary SS had anti-ribosomal P antibodies. There was no correlation between nonfocal CNS disease, including psychosis or severe depression, and the presence of anti-ribosomal P antibodies. Paired serum CSF samples from 34 SS patients with active CNS disease, including 6 with psychosis and 5 with severe depression, did not contain anti-ribosomal P.

In Sène et al. study [25], patients with pSS and PNS involvement and nonataxic sensory neuropathy were characterized by a higher age (57.5 ± 10.7 versus 48.7 ± 14.3 years; *P* = 0.007), more frequent CNS involvement (15% versus 2%; *P* = 0.04), and lower prevalence of ANA (60% versus 90%; *P* = 0.003), anti-SSA (Ro) (40% versus 72%; *P* = 0.009), anti-SSB (La) (15% versus 41%; *P* = 0.039), RF (37% versus 67%; *P* = 0.02), and hypergammaglobulinemia (35% versus 64%; *P* = 0.023). In multivariate analysis, nonataxic sensory neuropathy was associated with the presence of CNS involvement (OR, 17.0; *P* = 0.025) and ANA (OR, 0.07; *P* < 0.001).

In Delalande et al. study [23], fifty-six patients with pSS had CNS disorders, which were mostly focal or multifocal. Twenty-nine patients had spinal cord involvement (acute myelopathy (*n* = 12), chronic myelopathy (*n* = 16), or motor neuron disease (*n* = 1)). The disease mimicked relapsing-remitting multiple sclerosis (MS) in 10 patients and primary progressive MS in 13 patients. CNS symptoms included seizures (*n* = 7), cognitive dysfunction (*n* = 9), and encephalopathy (*n* = 2). Fifty-one patients had PNS involvement: symmetric axonal sensorimotor polyneuropathy with a predominance of sensory symptoms or pure sensory neuropathy occurred most frequently (*n* = 28), followed by cranial nerve involvement affecting trigeminal, facial, or cochlear nerves (*n* = 16). Multiple mononeuropathy (*n* = 7), myositis (*n* = 2), and polyradiculoneuropathy (*n* = 1) were also observed. Anti-Ro/SSA or anti-La/SSB antibodies were detected in 21% of patients at the diagnosis of SS, more frequently observed in patients with PNS involvement than in those with CNS involvement (*P* < 0.01).

Alexander et al. [22] examined 49 SS patients (group I) for the potential relationship between anti-Ro(SS-A) antibodies and active CNS disease by double immunodiffusion. In group 2 with 52 SS patients and active CNS disease, the anti-SSA antibodies were detected by ELISA. This study showed that anti-Ro antibodies were positive in 48% of patients with CNS manifestations and serious complications compared to only 24% of all patients with pSS.

Pittock et al. [24] detected NMO-IgG in 5 of 14 patients (35.7%) with NMOSDs and SS/SLE and in 2 of 4 patients (50.0%) with NMO without SS/SLE (*P* = 0.59). SSA, SSA, ANA, and dsDNA antibodies were found in these patients but not NMO-IgG.

## 4. Discussion

In this first systematic review of both CNS and PNS involvement in* RA*,* SLE*, and* pSS*, our results indicate that CNS complications (9–92%) are much more frequent than those of PNS (8–66%) across the various studies (total of 2338 patients). These findings are compatible with previous results in the literature. In this review, the majority of reported studies attributable in SLE CNS manifestations range from 13 to 92% of the events compared to PNS in 8–56%. The most frequent NP syndromes were headache, mood disorders, and seizures which are consistent with previous meta-analysis [33]. Ours finding are also consistent with previous studies where SLE CNS manifestations range from 33% to 75% [34, 35]. In SS studies, CNS manifestations range between 9 and 44% and PNS ranged between 24 and 68%, which agree with previous studies [36]. In this review, the frequency of PNS (mononeuritis) in RA patients is 18% lower as compared to other study, while for entrapment neuropathy (6% in our patients) it varies from 4% to 54.6% [37, 38].

Overall prevalence is seen to vary widely among the studies and is mainly attributed to a number of factors including bias in selection of patients for study, disease duration, and lack of uniformity in diagnostic criteria. With this in mind, we sought to expand the probability of specific manifestations by using established criteria and double-reading (by a certified neurologist), and excluding uncertain cases.

Among the most interesting points of this review is the correlation between CNS/PNS manifestations and immune activation markers (i.e., antinuclear antibodies ANA, anti-Ro/SSA, anti-La/SSB, RF, and hypergammaglobulinemia). Anti-Ro and anti-La seem to be less frequent in pSS patients with neurological involvement (40%) compared to patients without neurological manifestations (60% of positivity), so, it is necessary for more markers are necessary in pSS to better classify subpopulations of patients with neurological involvement [39]. Jamilloux et al. [26] claimed that the implication that the immunological profile (as potential serological markers) may only be a signature related to distinct neurological rather than a reflection of the pathogenic mechanism could not be dismissed. Nonetheless, given the apparent association with Raynaud's phenomenon, cutaneous vasculitis, and renal involvement, they proposed that sensorimotor neuropathy or mononeuritis multiplex originates from immunovascular injury. Contrary to other studies, Jamilloux et al. showed that sensory ganglionopathy is associated with lymphocytic infiltration and not cryoglobulinemia. In the same manner, Mellgren et al. showed that sensory ganglionopathy in pSS was related to lymphocyte infiltration of the dorsal root ganglia. In addition, Gono et al. reported optic neuropathy in one patient with SS and NMO (Aquaporin Ab positive) and MS, suggesting the implication of more mechanisms associated with the pathogenesis of neurological involvement in SS rather than the previously described vasculopathy. In the same context, Pittock et al. concluded that NMO disorders with seropositive findings for NMO-IgG occurring with SS/SLE or non-organ-specific autoantibodies is an indication of coexisting NMO rather than a vasculopathic or other complication of SS/SLE. Florica et al. observed that the group of SLE-related PN patients had more frequent CNS involvement and a higher score of disease activity, indicating that the immune response shows a preference toward neurological tissue. This is the first large study solely focused on peripheral neuropathy in SLE which suggests an autoimmune etiology for PN manifestations in SLE. It remains undetermined whether any of these clinical characteristics could predict the onset of PNS or/and CNS in either SS or SLE or other connective tissue disorders.

Several specific autoantibodies are associated with NPSLE (antiphospholipid, serum anti-ribosomal P antibodies, ANA, and antibodies to dsDNA). The presence of antiphospholipid antibodies is significantly associated with cerebrovascular disease and cognitive dysfunction, whereas serum anti-ribosomal P antibodies are strongly associated with psychosis and depression in SLE as discussed in this review [9, 10, 13, 20]. These findings are consistent with one multicenter study [40]. Moreover, the presence of cutaneous vasculitic lesions was significantly associated with NPSLE in the study by Kasitanon et al., rather than thrombocytopenia, as suggested by other studies [41]. According to Chiewthanakul et al., cutaneous vasculitis was only marginally related to active CNS involvement. Notably, in this study, neither ANA and antibodies to dsDNA nor anti-ribosomal P antibodies correlated with NP manifestations. In the same line is Spezialetti et al. study [21]. It seems that the independent associations of such manifestations with NPSLE have been difficult to study so far. This is partly explained by the difficulty to conduct studies with an adequate sample size.

However, Andonopoulos et al. failed to find a statistically significant correlation between the observed PNS pathology and any other clinical or immuno/serologic data. In the absence of any obvious reason, it is tempting to ascribe PNS symptoms to vasculitis of the vasa nervorum, but the data provided cannot fully support this hypothesis. The classical severe picture of a patient presenting with mononeuritis multiplex, associated with vasculitis in other organs, as already mentioned, leaves little doubt as to the pathogenesis of the neurologic syndrome. However, no patient has been reported to exhibit additional features of CNS disease that could be clinically detected.

Similarly, Sivri and Güler-Uysal found no correlation between mononeuritis and the immunological data in the serum of RA patients (i.e., RF), which is in contrast with the finding by other study [42]. This could be explained by the implication of a number of multiple factors in determining the clinical signs of disease (i.e., nonsteroidal anti-inflammatory drugs, NSAIDs). The single center, tertiary referral center-based patient population and small number of subjects might also explain the result.

Several limitations which compromised their external validity were identified in the specific studies, mainly by their authors. In terms of reporting complications, methods varied considerably. In this review, incidence was determined from studies of varying sample sizes across multiple age groups. Data collection was retrospective [14, 19, 29, 32]. Hence, patient assessment might have been incomplete, rendering it difficult to analyze details of NPSLE syndromes [13] or missing PN initial events [20]; secondly, other CNS manifestations, such as headaches, anxiety, cognitive dysfunction, and autonomic disorders, may have been missed; thus, their prevalence has been underestimated [19, 30]. Finally, the cause of death by autopsy was not always performed; thus, authors could not conclude whether death was related to SLE-mediated organ dysfunction or other diseases [15]. Perhaps due to methodological limitations it was not possible to determine if the associations between medication use with the occurrence of NP events and renal disease were independent of each other [9].

Notwithstanding, this review is not without its limitations. There are a small number of studies available for analysis. One limitation of this review is that a limited number of databases were searched. Consequently, there is always the possibility that we overlooked studies in other databases published before 1980. In addition, there is always the possibility of publication bias due to underreported negative results and grey literature. However, the use of fewer search limits increases the sensitivity of the search method.

## 5. Conclusions

Despite the abovementioned limitations, this systematic review provides foundation for further research on the pathophysiology of immune system among the vasculature of the CNS and PNS. It is still a matter of debate whether the CNS and PNS manifestations of RA, SLE, and pSS are direct effect of these diseases or secondary such as side effects of corticosteroids and other immunosuppressive therapies or multiple systemic organ dysfunction (i.e., drug or SLE-mediated multiorgan induced psychosis). Τo date, no laboratory test, that is, autoantibodies, has been shown as pathognomonic neither for CNS nor for PNS involvement in these connective tissue disorders. Prospective multicenter studies are warrant for further confirmations.

## Figures and Tables

**Table 1:** Most frequent autoantibodies in nervous system involvement in connective tissue diseases SLE, SS, and RA.

Connective tissue diseases	Autoantibodies (% of positive patients, clinical association)
SLE	ANA Anti-dsDNA (nephritis, disease activity) Anti-ribosomal P (disease activity, NPSLE) Anti-Sm (highly specific for SLE) Anti-Ro (DD Sjögren's syndrome) Anti-La (DD Sjögren's syndrome) Antiphospholipid (thrombosis) AQP-4 (overlap NMO spectrum)

SS	ANA Anti-Ro (DD SLE) Anti-La (DD SLE) RF (lymphoma) Cryoglobulins (vasculitis, lymphoma) AQP-4 (overlap NMO spectrum)

RA	ANA

SLE: systemic lupus erythematosus, NP: neuropsychiatric, SS: Sjögren's syndrome, RA: rheumatoid arthritis, ANA: antinuclear antibodies, Anti-Sm: anti-Smith, Anti-Ro: Ro antigen, Anti-La: lupus anticoagulant, anti-dsDNA: anti-double-stranded DNA, RF: rheumatoid factor, DD: differential diagnosis, AQP-4: Aquaporin, and NMO: Neuromyelitis optica.

**Table 2:** Characteristics of the selected clinical trials focusing on PNS and CNS complications of SLE, pSS, and RA.

Authors/year	Type of study	Sample characteristics (sex, years in median or mean ± SD)	Type of CNS involvement % (*n*) of patients	Type of PNS involvement	Clinical associations with immunological profiles
SLE patients^*∗*^					

Schneebaum et al., 1991 [9]	Cohort	269 patients with SLE	Depression 88% (*n* = 8), 45% psychosis (*n* = 29)	NM	The serum level of anti-P antibodies correlates with the activity of psychiatric disease

Isshi and Hirohata, 1998 [10]	Cohort	87 SLE patients	Psychosis 39% (*n* = 34), nonpsychotic CNS lupus 29,9% (*n* = 26)	NM	Serum anti-P levels were significantly elevated in patients with lupus psychosis compared with those with non-CNS SLE or those with nonpsychotic CNS lupus

Tzioufas et al., 2000 [11]	Cohort	28 patients with NPSLE	Psychiatric disorders 25% (*n* = 7), grand mal seizures 14,2% (*n* = 4), focal signs 46,4% (*n* = 13)	NM	Overall prevalence of anti-P antibodies in active CNS disease patients was statistically and significantly higher, as compared to unselected SLE patients

Kasitanon et al., 2002 [12]	Cohort	91 patients with NPSLE (90 F, 1 M, 30,7 ± 10,9)	Seizures 58,3% (*n* = 53), psychosis 14,3% (*n* = 13), acute confusion state 12% (*n* = 11), cerebral infraction 2,2% (*n* = 2), abnormal consciousness 6,6% (*n* = 6), transverse myelitis 6,6% (*n* = 6), aseptic meningitis 2,2% (*n* = 2)	MM 3,3% (*n* = 3), polyneuropathy 2,2% (*n* = 2)	Patients with NPSLE had significantly more cutaneous vasculitis and less arthritis than those without NPSLE

Sanna et al., 2003 [13]	Cohort	185 patients with NPSLE	Headache 24% (*n* = 78), CD 14.5% (*n* = 47), mood disorders 16.7% (*n* = 54), cognitive disorders 10.8% (*n* = 35), seizures 8.3% (*n* = 27), psychosis 7.7% (*n* = 25), anxiety 3.7% (*n* = 24), acute confusional state 3.7% (*n* = 12)	NM	The presence of aPL was associated with NP manifestations

Chiewthanakul et al., 2012 [14]	Cohort	97 patients with NPSLE (84 F, 13 M, 35.1 ± 11.7)	Seizures 33% (*n* = 32), CD 23,7% (*n* = 23), psychoses 24% (*n* = 23), myelopathy 6,2% (*n* = 6), headaches 2% (*n* = 2), mood disorders 1% (*n* = 1)	13 patients with polyneuropathy 77% (*n* = 10), GB 7,7% (*n* = 1), mononeuropathy 7,7% (*n* = 1), cranial neuropathy 7,7% (*n* = 1)	ANA and antibodies to dsDNA did not correlate with NP manifestations

Závada et al., 2013 [15]	Cohort	50 patients with NPSLE (5 M, 45 F 43 years (±16))	Cognitive disorder 50% (*n* = 25), mood disorder 28% (*n* = 14), CD 26% (*n* = 13), headache 26% (*n* = 13), seizure 22% (*n* = 11), psychosis 16% (*n* = 8), aseptic meningitis 4% (*n* = 2)	Polyneuropathy 8% (*n* = 4), cranial neuropathy 6% (*n* = 3), mononeuropathy 4% (*n* = 2), transverse myelitis 2% (*n* = 1)	NMO-IgG/AQP4-Ab in NPSLE were present only in a patient with TM and were not detectable in NPSLE patients with other neurological manifestations

Hanly et al., 2004 [16]	Cohort	111 patients with SLE (96 F, 15 M, 44.7 ± 1.2 years)	Headaches 9% (*n* = 10), CD 3,6% (*n* = 4), mood disorders 3,6% (*n* = 4), cognitive dysfunction 2,7% (*n* = 3), acute confusional state 2,7% (*n* = 3), psychoses 2,7% (*n* = 3), seizures 0,9% (*n* = 1), anxiety 0,9% (*n* = 1), aseptic meningitis 0,9% (*n* = 1)	Cranial neuropathy 1,8% (*n* = 2), polyneuropathy 1,8% (*n* = 2)	No correlations were found

Kluz et al., 2007 [17]	Cohort	15 F mean age: 38.33 ± 11.02 years with SLE	Organic brain syndrome 13,3% (*n* = 2), cranial nerve disorder 6,6% (*n* = 1), headache 6,6% (*n* = 1), seizures 6,6% (*n* = 1)	NM	CNS complications were associated with aPL antibodies in patients with severe disease activity and microangiopathic complications compared with those with less active disease

Briani et al. 2009 [18]	Cohort	85 SLE patients (NM sex and mean age)	Headache 41,2% (*n* = 35), CD 12,3% (*n* = 11), epilepsy 11,8% (*n* = 10), psychiatric disorders 3% (*n* = 6), myelopathy 2,4% (*n* = 2)	Symmetric polyneuropathy 20% (*n* = 17), mononeuropathy 15,3% (*n* = 13), median nerve 3,6% (*n* = 3) sciatic nerve 3,6% (*n* = 3) involvement, MM 2,4% (*n* = 2)	Abs to ribosomal P proteins are associated with psychosis and MM

Florica et al., 2011 [19]	Case-control (retrospective)	207 SLE patients, F 86.3% (125 SLE-related PN 35.2 ± 14.4 years and 82 non-SLE-related PN 38.6 ± 15.4)	NM	PM 11,1% (*n* = 23), cranial neuropathy 12,5% (*n* = 26), MM 9,2% (*n* = 19), CIDP 0,9% (*n* = 2)	There was no significant difference in lupus serology such as antinuclear antibody, anti-double-stranded DNA antibody, and antibodies to the extractable nuclear antigens between the two groups

Hirohata et al., 2014 [20]	Cohort	72 patients with NPSLE (49 with diffuse NPSLE 38.3 ± 14.4, 23 with neurological syndromes or peripheral neuropathy 42.0 ± 15.2) 32 M, 50 F	Diffuse NPSLE: acute confusional state 38,7% (*n* = 19), anxiety 6,1% (*n* = 3), cognitive disorder 6,1% (*n* = 3), psychosis 14,2% (*n* = 7); focal NPSLE: headache 8,7% (*n* = 2), movement disorder 8,7% (*n* = 2), seizure 8,7% (*n* = 7), aseptic meningitis 4,3% (*n* = 1), demyelinating syndrome 4,3% (*n* = 1)	Polyneuropathy 4,3% (*n* = 1)	Anti-Sm and anti-RNP in CSF and sera were elevated in NPSLE compared with non-SLE control

SS patients					

Spezialetti et al., 1993 [21]	Cohort	77 patients with pSS	Severe depression 13% (*n* = 10), psychosis 8% (*n* = 6), cognitive dysfunction 69% (*n* = 54)	NM	No correlation between CNS diseases, including the presence of anti-ribosomal P antibodies

Alexander et al., 1994 [22]	Cohort	Group 1: 52 SS patients Group 2: 49 patients	Group 1: focal CNS disease 60% (*n* = 26/43), nonfocal CNS disease 22% (*n* = 2/9) Group 2: focal CNS disease 63% (*n* = 19/30), nonfocal CNS disease 37% (*n* = 7/19)	Not available	Anti-Ro antibodies were positive in 48% of patients with CNS compared to only 24% of all patients with pSS

Delalande et al., 2004 [23]	Cohort	82 patients (65 F, 17 M 48,6 years)	Seizures 8,5% (*n* = 7), cognitive dysfunction 11% (*n* = 9), encephalopathy 2,4% (*n* = 2), optic neuropathy 15% (*n* = 13), MS 28% (*n* = 23)	SMN 34,1% (*n* = 28), cranial neuropathy 19,5% (*n* = 16), MN 8,5% (*n* = 7), myositis 2,4% (*n* = 2), polyradiculoneuropathy 1,2% (*n* = 1)	Anti-Ro/SSA or anti-La/SSB antibodies were more frequently observed in patients with PNS involvement than in those with CNS involvement

Pittock et al., 2008 [24]	Cohort	14 patients with SS/SLE with neurological manifestations	NM	NM	SSA, SSA, ANA, and dsDNA antibodies were found in these patients but not NMO-IgG

Sène et al., 2011 [25]	Cohort	120 patients with pSS (106 F, 14 M 50.4 ± 14.0)	NM	SMN 23% (*n* = 7), ASN 10% (*n* = 3), NSN 67% (*n* = 20)	Patients with NSN with lower prevalence of ANA (60% versus 90%; *P* = 0.003), anti-SSA (Ro) (40% versus 72%; *P* = 0.009), anti-SSB (La) (15% versus 41%; *P* = 0.039), RF (37% versus 67%; *P* = 0.02), and hypergammaglobulinemia (35% versus 64%; *P* = 0.023)

Jamilloux et al., 2014 [26]	Cohort	420 patients with pSS (377 F, 43 M, 53.6 ± 14.8)	Acute ischemic stroke-like symptoms 1,4% (*n* = 6), dysarthria 0,2% (*n* = 1), seizures 0,2% (*n* = 1), cerebellar ataxia 0,4% (*n* = 2) central neuronitis 0,4% (*n* = 2), acute encephalopathy 2,1% (*n* = 9), cognitive impairment 0,4% (*n* = 2), aseptic meningitis 0,2% (*n* = 1), transverse myelitis (*n* = 12), optic neuritis 1,2% (*n* = 5), MS-like syndrome 1,2% (*n* = 5), cerebral venous thrombosis 0,2% (*n* = 1)	SMN 0,6% (*n* = 25), MM 0,7% (*n* = 3), SN 4,5% (*N* = 19), SGN 2,1% (*n* = 9), DPN 0,2% (*n* = 1), cranial neuropathy 1,9% (*n* = 8)	Patient with SN had more frequent cryoglobulinemia and lymphopenia (*P* < 0.05) but lower prevalence of anti-Ro/SSA antibodies and hypergammaglobulinemia (*P* < 0.05)

Morreale et al. 2014 [27]	Cohort	120 patients (12 M, 108 F; 58.3 ± 14.2 years)	Headache 46.9% (*n* = 30), cognitive disorder 44.4% (*n* = 28), mood disorders 38.3% (*n* = 6)	NM	Headache, cognitive disorders, and psychiatric symptoms were significantly associated with anti-SSA

RA					

Sivri and Güler-Uysal, 1999 [28]	Cohort	33 RA patients (28 F, 5 M; 46.7 ± 13.7 years)	NM	6% (*n* = 2) carpal tunnel syndrome, 18% (*n* = 6) MM	No correlation between neuropathy and RF

*n*: number of patients, F: female, M: male, SD: standard deviation, pSS: primary Sjögren syndrome, RA: rheumatoid arthritis, RF: rheumatoid factor, NMO: Neuromyelitis optica, PN: peripheral neuropathy, SMN: sensorimotor neuropathy, ASN: ataxic sensory neuropathy, NSN: nonataxic sensory neuropathy, SFN: small fiber neuropathy, PN: peripheral mononeuropathy, MM: mononeuritis multiplex, SN: sensory neuropathy, SGN: sensory ganglionopathy, DPN: demyelinating polyradiculoneuropathy, ANS: autonomous nervous symptoms, PNS: peripheral nervous system, CNS: central nervous system, CD: cerebrovascular disease, MS: multiple sclerosis, NP: neuropsychiatric disease, SLE: systemic lupus erythematosus, aPL: antiphospholipid antibodies, CIDP: chronic inflammatory demyelinating polyradiculoneuropathy, AIDP: acute inflammatory demyelinating polyradiculoneuropathy, focal CNS disease: one or more fixed focal clinical deficits of brain or spinal cord with or without psychiatric or cognitive dysfunction, nonfocal CNS disease: psychiatric or cognitive dysfunction or both without focal neurologic deficit, and NM: not mentioned; ^*∗*^according to the 1999 ACR definition of NPSLE.
