# Corrigendum to “A Systematic Review of Peripheral and Central Nervous System Involvement of Rheumatoid Arthritis, Systemic Lupus Erythematosus, Primary Sjögren's Syndrome, and Associated Immunological Profiles”

**DOI:** 10.1155/2016/9854813

**Published:** 2016-04-07

**Authors:** Anastasia Bougea, Evangelos Anagnostou, Konstantinos Giatas, George Paraskevas, Nikolaos Triantafyllou, Evangelia Kararizou

**Affiliations:** 1st Department of Neurology, University of Athens Medical School, Eginition Hospital, Athens, Greece

In the review article titled “A Systematic Review of Peripheral and Central Nervous System Involvement of Rheumatoid Arthritis, Systemic Lupus Erythematosus, Primary Sjögren's Syndrome, and Associated Immunological Profiles” [1], the author list contained an error. The first and last names of the third and fourth authors were reversed. The author list is presented correctly in the front matter of this correction.

## References

[1] Bougea A., Anagnostou E., Konstantinos G., George P., Triantafyllou N., Kararizou E. (2015). A systematic review of peripheral and central nervous system involvement of rheumatoid arthritis, systemic lupus erythematosus, primary Sjögren’s syndrome, and associated immunological profiles. *International Journal of Chronic Diseases*.

